# Intraoperative Optical Coherence Tomography (OCT)-Guided Femtosecond Laser-Assisted Descemet Membrane Endothelial Keratoplasty (iFAD)

**DOI:** 10.3390/bioengineering11121192

**Published:** 2024-11-25

**Authors:** Joshua Lim, Mohammed M. Abusayf, Yu-Chi Liu, Jodhbir S. Mehta

**Affiliations:** 1 Singapore National Eye Center, Singapore 168751, Singapore; joshua.lim@singhealth.com.sg (J.L.); mmabuseif@gmail.com (M.M.A.); liu.yu.chi@snec.com.sg (Y.-C.L.); 2 Singapore Eye Research Institute, Singapore 169856, Singapore; 3 Department of Ophthalmology, College of Medicine, King Saud University, Riyadh 11362, Saudi Arabia; 4 Ophthalmology and Visual Sciences Academic Clinical Programme, Duke-NUS Medical School, Singapore 169857, Singapore

**Keywords:** femtosecond laser, intraoperative optical coherence tomography, corneal surgery

## Abstract

We describe retrospectively the indications and outcomes of nine patients who present with varying degrees of deep posterior stromal scarring or endothelial failure following deep anterior lamellar keratoplasty (DALK). These patients underwent a surgical strategy coined Intraoperative Optical Coherence Tomography Guided Femtosecond Laser-Assisted Descemet Membrane Endothelial Keratoplasty (iFAD). This strategy can be used to address suboptimal visual outcomes following primary DALK. Patients undergoing iFAD first had femtosecond laser-assisted trephination of the underlying posterior lamella using the liquid keratoplasty system on the Femto LDV Z8 platform (Zeimer Ophthalmic Systems AG, Port, Switzerland). A Descemet Membrane Endothelial Keratoplasty (DMEK) graft was subsequently transplanted in place of the removed lamella. Out of nine cases, major indications for seven included post-infective, blepharokeratoconjunctivitis (BKC) and deep stromal scarring related to stromal dystrophy. The remaining two had endothelial failure following primary DALK. The main benefits of this surgical approach are (1) Utilization of integrated real-time iOCT on the femtosecond laser platform allows for precise calibration of a wide range of vertical posterior trephination depths (96–329 microns) from the endothelial surface. (2) Femtosecond laser trephination utilizing a non-applanation liquid interface preserves trephination geometries and maximises precision-based surgical outcomes. (3) iFAD is a viable and straightforward technique for surgeons addressing patients who might otherwise require complex surgery to address residual deep stromal scars post-DALK.

## 1. Introduction

Prior to the advent of lamellar keratoplasty techniques, corneal opacities in the stroma were traditionally addressed through penetrating keratoplasty (PK). While PK was effective in improving visual clarity, numerous reports over the years have highlighted the multitude of intraoperative and postoperative complications associated with this procedure [1]. Foremost among the feared intraoperative complications is the occurrence of expulsive suprachoroidal haemorrhage following the removal of the recipient’s corneal button [2,3]. Additionally, a lifetime risk of postoperative graft rejection necessitates an equally lengthy treatment with topical immunosuppression, leading to secondary complications like glaucoma and cataract formation [4,5]. The landscape of corneal surgery has since been substantially transformed by the introduction of lamellar procedures [6,7].

Lamellar procedures, particularly anterior lamellar keratoplasty (ALK), have evolved significantly since their inception in the early 1990s primarily toward addressing stromal opacities. Anwar and Teichmann’s introduction of the deep anterior lamellar keratoplasty technique (DALK) in 2002 was a landmark step, allowing stromal dissections to extend deeper toward Descemet’s membrane (DM) [8]. This innovation led to a reduced long-term risk of complications such as graft rejection. Lamellar grafts also allow for a tectonically stronger eye and visual outcomes often matching or surpassing even that which was attainable through PK [7,9,10]. Continuous improvements and refinements in surgical techniques have affirmed the safety and efficacy of this procedure [7,11,12], and the advantages of ALK over PK have been well documented in the scientific literature [11,12,13].

Despite clear benefits in long-term surgical outcomes, DALK remains behind PK as a preferred surgical option for significant proportions of surgeons around the world [14]. This is in part surgically due to the procedure being technically demanding, with a steep learning curve [15,16]. A significant issue that can follow DALK is the development of deep residual stromal scarring. This can arise from difficulties encountered during dissection or from remnant folds left behind in the deep layers due to original underlying pathology (e.g., herpetic stromal keratitis (HSK), and post-hydrops scars). Subsequent chronic endothelial compromise may also result in deep stromal scarring. Various strategies have been utilized to address this post-operative issue, ranging from conservative management to redoing DALK with additional stromal bed dissection. Some cases even require repeat surgery with a PK [17,18].

The significant decision to convert to a PK should be weighed carefully. The main considerations are that of long-term graft survival and PK rejection, especially in young patients or high-risk cases, including that of post-infections, or those with extensive stromal vascularization [17]. Advancements in surgical technology over the years, especially in the realm of femtosecond lasers have provided some solutions to the above challenges. Developed initially for cataract and refractive surgery, femtosecond lasers have since expanded their applications into keratoplasties [19,20]. Numerous reports detail improved functional and histological outcomes in femtosecond laser-assisted PKs and DALKs. The benefits of femtosecond lasers in keratoplasties stem from the ability to produce precise dimensional trephinations between donor and host corneal interfaces, in the case of full-thickness transplants, or the ability to create a stromal tunnel precisely to the level of pre-Descemetic space, in lamellar dissections [20,21,22,23]. In this report, we demonstrate through nine cases how the precision afforded by combining both intraoperative optical coherence tomography (iOCT) with the use of femtosecond laser can provide a novel strategy approaching challenging cases with residual scarring following DALK.

## 2. Methods

We conducted a prospective study of patients undergoing iFAD for residual deep stromal scarring following DALK, at the Singapore National Eye Center between January 2019 and March 2023. Informed consent was obtained from all subjects to participate in this study, which followed the principles of the Declaration of Helsinki. Surgery was performed by an experienced corneal surgeon (J.S.M.). Summary demographics of the nine cases can be found in Table 1.

### Surgical Procedure

**Figure 1:**
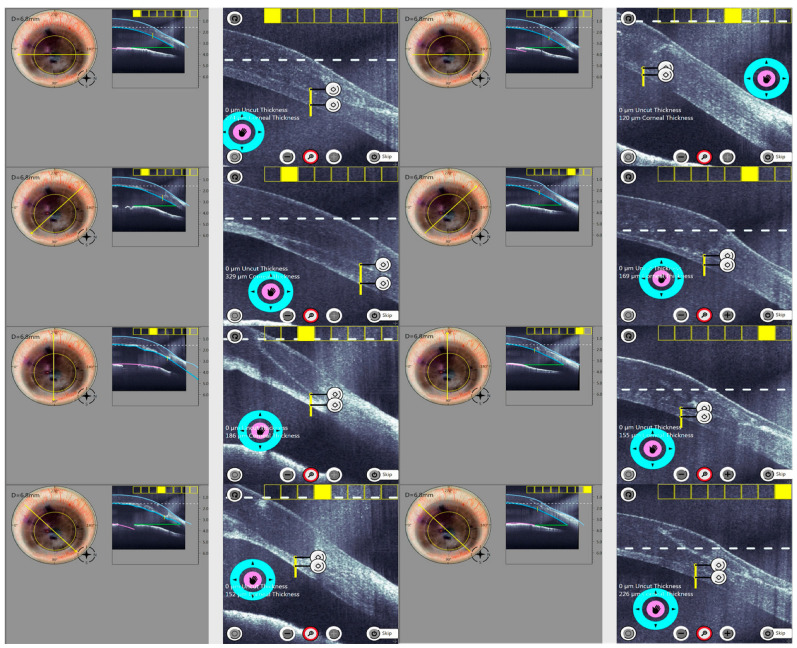
Intraoperative Optical Coherence Tomography (iOCT) pre-trephination phase. These stills were captured over all principal sectors demonstrating the process of programming vertical trephination side cut depths into the host cornea. With the aid of the iOCT, the interface between the original overlying DALK and the underlying posterior lamella can be observed. Precise trephination depths can be subsequently programmed up to the level of the DALK–posterior lamella interface.

**Figure 2:**
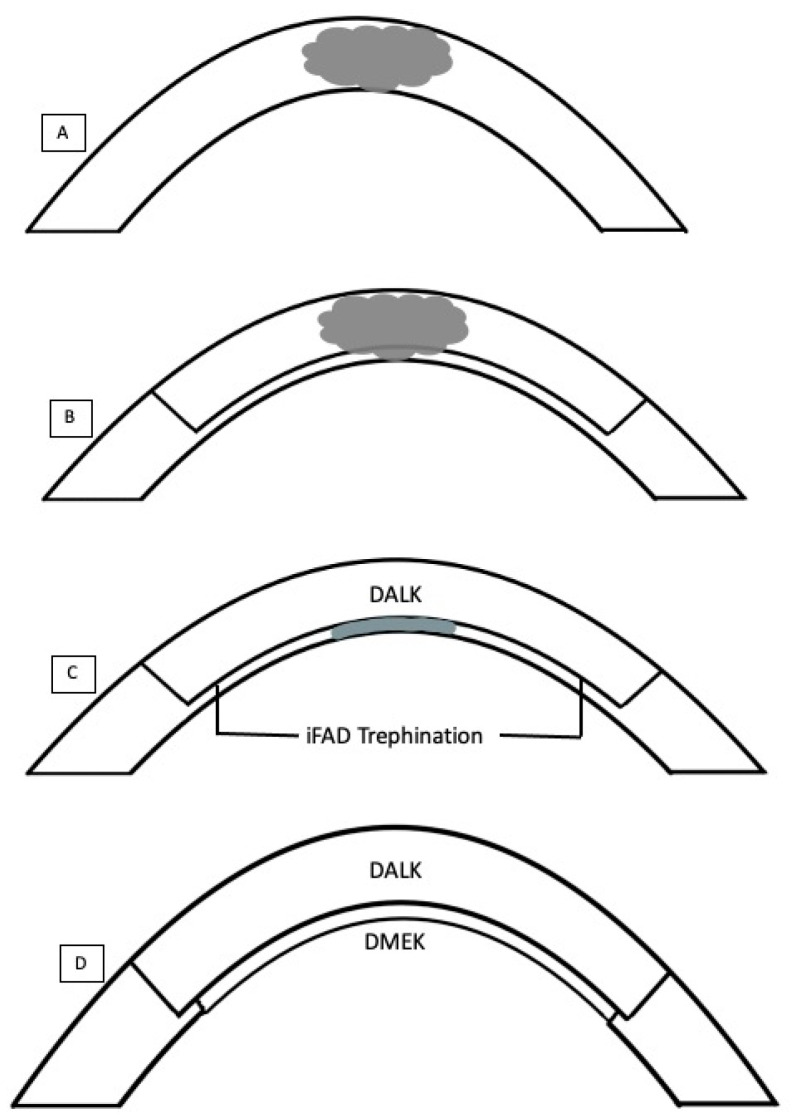
Schematic of the operative strategy. (**A**) Patient with a pre-existing deep stromal scar. (**B**) Initial anterior lamellar keratoplasty (ALK) is performed to optimize the visual outcomes via manual deep anterior lamellar keratoplasty (DALK). (**C**) Due to remnant deep stromal scarring obstructing the visual axis, iFAD using the femtosecond laser can be applied. A femtosecond laser is utilized to create a posterior trephination up to the posterior edge of the initial DALK graft (iFAD Trephination). (**D**) A DMEK graft is slotted into the area which previously underwent trephination. The diameter of this graft is sized slightly smaller than the trephined diameter to reduce the risk of graft detachment post-operatively.

**Figure 3:**
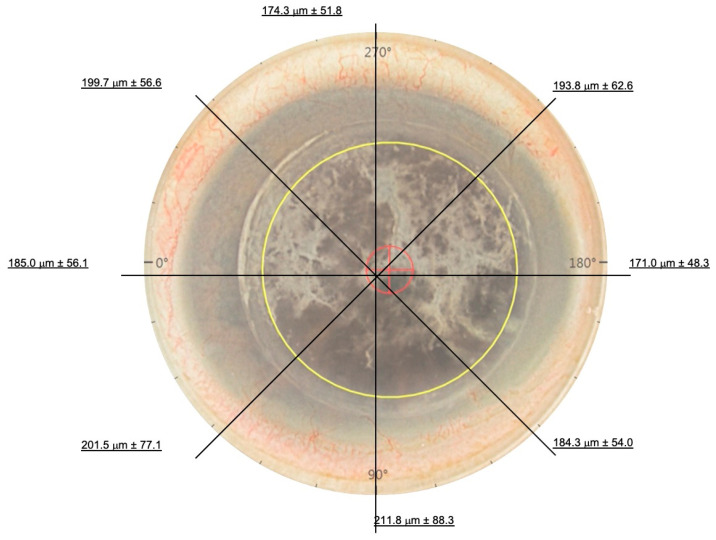
Figure illustrating the mean femtosecond laser trephination depths at the cardinal points of trephination. The cardinal points correspond to those seen in Figure 1.

All surgeries were performed under general or local anaesthesia with sedation. The iFAD procedure was performed with the Ziemer Z8 Femto LDVTM system (Ziemer Ophthalmic Systems, Port, Switzerland) on the liquid keratoplasty program. All nine cases had undergone previous DALK prior to iFAD. AS-OCT scans (Optovue, Fremont, CA, USA) were first reviewed pre-operatively in the clinic to estimate and pre-visualize the depth and size of vertical trephination. Intraoperatively, the femtosecond laser trephination diameter was deliberately undersized in reference to the overlying DALK. Following standard suction ring docking onto the eye, 3–5 mL of balanced salt solution was used to create a fluid–cornea interface. The Ziemer Z8 Femto LDVTM is different from other femtosecond laser systems in that it features a non-applanation interface. The fixed laser parameters used were: side cut angle of 90°, posterior safety distance of 0.7 mm, anterior corneal offset of 0 μm, posterior corneal offset of 200 μm, cut speed of 50 mm/s and laser power between 150–160% [24,25,26]. Variable parameters included trephination diameters inside the overlying DALK and vertical side cut depths. (Table 1) The in-built intraoperative OCT (iOCT) aided the surgeon in the centration of the trephination and in the adjustment of the vertical side cut thickness. Side cuts were programmed intraoperatively to encompass the trephination up to the anterior-most extent of the graft–host junction, and were programmed in 30-degree intervals around the cornea recipient. Intraoperative iOCT still demonstrates this process in Figure 1. The laser was fired to create the trephination. Cases that had visually significant cataracts underwent phacoemulsification following trephination to ensure anterior chamber stability throughout trephination. A reverse Sinskey hook (product number E3119, Bausch + Lomb Storz Ophthalmic Instruments, Heidelberg, Germany) was used to dissect the trephined posterior lamella. Any adhesions, if present, between the lamella and posterior stroma were plicated and released. The lamella was removed en bloc through a 2.65 mm limbal incision placed temporally. An inferior surgical peripheral iridectomy was created in all cases. A pre-stripped Descemet Membrane Endothelial Keratoplasty (DMEK) graft obtained from the Singapore Eye Bank was inserted using a previously described standard pull-through ‘endothelium-in’ technique, utilizing a CORONET DMEK EndoGlide (Network Medical Products, North Yorkshire, UK) [27,28] and pulled through with microforceps (product number AE-4962, ASICO LLC, ASICO, LLC. Parsippany, NJ, USA). The DMEK graft circumference was sized 0.1–0.4 mm smaller than the initial femtosecond laser circumference The graft was allowed to open spontaneously in the correct orientation [27,29]. Attachment onto the recipient stroma was achieved with a full anterior chamber of air or a non-expansile Sulfur Hexafluoride (SF6, 20%) fill [28]. A schematic is provided to illustrate the surgical steps in Figure 2. Patients were postured face up overnight, and were started on topical antibiotics (Levofloxacin 0.5%; Santen, Osaka, Japan) and tapering topical steroids (Pred. Forte 1%; Allergan, Dublin, Ireland). Patients were followed up regularly in the outpatient cornea service. Data collected included demographics of all patients, pre-, 6 and 12 months post-operative visual acuity, number of prior DALK surgeries before the iFAD procedure, diameters of the pre-existing DALK graft, trephined posterior lamellar and subsequent DMEK grafts, in Table 1. A diagrammatic representation of the mean posterior vertical trephination depths along the principal meridians for all nine cases is shown in Figure 3.

## 3. Results

**Figure 4:**
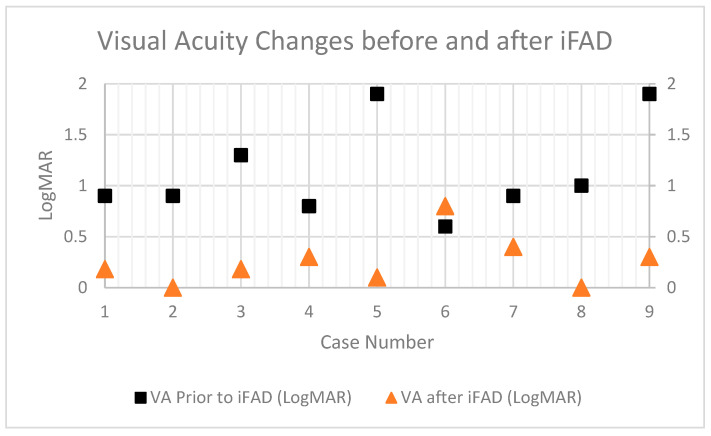
Graphical representation of visual acuities (VA) in LogMAR before and after iFAD. Square points represent VA pre-iFAD, and triangular points represent VA post-iFAD.

Included in this study were nine eyes of nine Asian patients with a mean age of 59.2 ± 25.1 years. Seven cases underwent iFAD to address residual deep stromal scars or recurrences in stromal pathology following initial DALK. Two cases had both deep stromal scars and endothelial decompensation requiring iFAD. Figure 4 summarizes visual acuity changes in the cases before and after iFAD. Short case vignettes are provided for the cases. (Additional images can be found in the Supplementary Materials).

### 3.1. Addressing Residual Deep Stromal Scars/Recurrence of Pathology

#### 3.1.1. Patient 1

**Figure 5:**
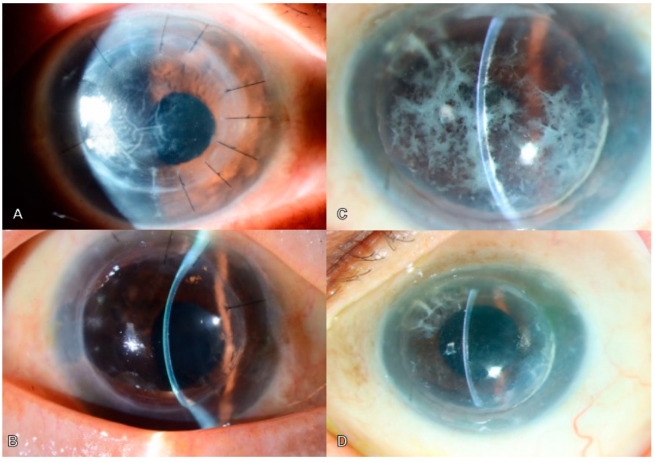
Representative photographs of cases 1 and 4. (**A**) Patient in case 1 with a significant and deep stromal scar following initial DALK. Deep stromal folds and undulations can be appreciated. (**B**) Following iFAD, optical clarity is improved over the visual axis. (**C**) Patient in case 4 with granular dystrophy. The patient had a DALK performed years prior; however, the dystrophy had recurred mainly in the deep stromal layers. (**D**) Following iFAD, corneal clarity is much improved. A photo montage of the other cases can be found in the Supplementary Materials.

A 72-year-old female underwent an 8.0 mm manual optical DALK for corneal stromal scarring secondary to old herpes simplex stromal scar. She was noted to have deep residual posterior stromal folds within the visual axis, with 20/200 vision (LogMAR 1.00). (Figure 5A) Clinical exam showed a stable and clear anterior lamellar graft with deep, undulating posterior stromal folds 57–118 microns from the endothelial surface. She underwent iFAD 2 years after the initial DALK. Intraoperatively, deep posterior stromal folds were noted in the residual recipient stromal bed. The femtosecond laser trephined a 7.6 mm posterior lamella at 150% power, which was subsequently peeled from the overlying DALK graft. A 7.5 mm DMEK graft was inserted using an Endo In technique, via an Endoglide [28]. Postoperatively, a persistent inferior DM detachment was noted up to 2 weeks, and she underwent repeat intracameral injection of 20% SF6. At post-operative month 6 (POM6), the DMEK graft remained attached and clear, with an overlying compact and clear anterior lamellar graft and complete clearance of posterior stromal folds. (Figure 5B) The patient achieved a best corrected visual acuity (BCVA) of 20/30 (LogMAR 0.18).

#### 3.1.2. Case 2

A 55-year-old male had a longstanding herpetic stromal scar. A dense stromal scar was noted centrally and inferiorly, with undulating deep posterior folds and stromal vascularization, correspondingly imaged on AS-OCT. Corneal thickness ranged from 732–845 microns. Vision was 20/160 (LogMAR 0.90) corrected to 20/63 (LogMAR 0.50) by pinhole. This was deemed a high-risk PK due to extensive corneal vascularization, and as such he underwent 8 mm manual DALK. Post-operatively, deep fixed folds were noted centrally and inferiorly at the graft host junction. Pinhole visual acuity was 20/100 (LogMAR 0.70). AS-OCT demonstrated deep posterior folds 112–225 microns from the endothelium. Four months post DALK, he underwent iFAD combined with phacoemulsification, with a 7.7 mm posterior trephination at 150% power, followed by a 7.5 mm donor DMEK graft. He achieved the best corrected vision of 20/20 (LogMAR 0.00) at POM8.

#### 3.1.3. Case 3

An 18-year-old female underwent two consecutive manual DALK within a period of 3 years due to blepharokeratoconjunctivitis-related stromal scarring, central corneal thinning and perforation. She was lost follow-up and returned 3 years after with extensive stromal vascularization. Like case 2, this was a high-risk PK, and as such, she underwent a third 7.75 mm DALK. The overlying DALK subsequently achieved stability; however, it was left with a diffuse DM scar with a visual acuity of 20/400 (LogMAR 1.3) corrected to 20/125 (LogMAR 0.8) on the pinhole. AS-OCT demonstrated a DM scar of 89–199 microns from the endothelium. She subsequently underwent iFAD with a 7.40 mm trephination at 150% power, followed by a 7.00 mm DMEK. Her final BCVA was 20/32 (LogMAR 0.2).

#### 3.1.4. Case 4

An 83-year-old male with a history of pituitary macroadenoma and advanced normal tension glaucoma (NTG) underwent a 7.5 mm manual optical DALK for stromal deposits secondary to Type 2 Granular Corneal Dystrophy (GCD). His BCVA post-DALK was 20/50 (LogMAR 0.4) corrected to 20/25 (LogMAR 0.1). There were eventual recurrences of the granular deposits in the deep stromal interface, with interval AS-OCT demonstrating hyperreflectivities 48–109 microns from the endothelial surface. This reduced his vision from 20/160 (LogMAR 0.9) to 20/125 (LogMAR 0.8) on the pinhole. (Figure 5C) He subsequently underwent iFAD with a 7.60 mm trephination at 150% power followed by a 7.25 mm DMEK graft. At post-operative month 8, the DMEK graft remained clear, with a reduction in the number of deep interface deposits. (Figure 5D) BCVA was 20/40 (LogMAR 0.3).

#### 3.1.5. Case 5

A 53-year-old female with a history of repeated contact lens-related infective keratitis underwent femtosecond laser-assisted optical HALK for an anterior scar [30,31]. Vision was 20/400 (LogMAR 1.3) pre-operatively. Femtosecond laser was utilized to trephine a 7.8 mm diameter anterior cap to a depth of 390 microns to encompass the scarred stroma. The anterior cap was then manually dissected to a depth of 220 microns and replaced with a 7.75 mm donor. BCVA improved to 20/80 (LogMAR 0.6) post-operatively. However, the patient had residual deep stromal scarring, 89–182 microns from the endothelial surface. She subsequently underwent iFAD combined with phacoemulsification, with a 7.50 mm trephination at 150% power, with a subsequent 7.25 mm DMEK graft. Post-operatively, she developed an inferior shallow DM detachment at week 1 which required rebubbling with intracameral 20% SF6. BCVA at POM4 was 20/25 (LogMAR 0.1).

#### 3.1.6. Case 6

An 82-year-old male with a history of advanced NTG, right eye cicatricial entropion, poor ocular surface and myopic macular degeneration underwent a 7.50 mm manual DALK for a stable interstitial keratitis stromal scar. Post-operative vision was affected by deep residual stromal folds, extending inferiorly and centrally over the visual axis. BCVA was 20/80 (LogMAR 0.6). AS-OCT demonstrated undulations and scarring up to 123 microns from the endothelial surface. He subsequently underwent iFAD with a 7.00 mm trephination at 150% power and a 6.75 mm DMEK graft. Post-operatively, a DM detachment was noted at day 4, requiring intracameral 20% SF6. At POM5 the DMEK graft remained attached. The best corrected vision remained at 20/160 (LogMAR 0.86) due to the underlying retinal pathology.

#### 3.1.7. Case 7

A 17-year-old female with a significant history of eye rubbing presented at a young age with bilateral superior corneal opacities. These were presumed old herpetic stromal scars. She was referred to with increasing left eye blurring of vision, secondary to significant posterior stromal thinning with ectasia. Initial vision was 20/160 (LogMAR 0.86). She underwent a 7.00 mm manual DALK, which was complicated by intraoperative micro-perforations in the areas of maximal thinning. Postoperatively, she was noted to have a double anterior chamber on day 4, and underwent intracameral air injection to reposition the dehisced DM. The DALK subsequently achieved stability with an attached DM. Visual recovery was limited, however, by the subsequent formation of a DM scar just off the visual axis. Visual acuity was 20/200 (LogMAR 1.0), and AS-OCT demonstrated a dense hyperreflective scar of 116–196 microns in thickness from the endothelium. In view of the significant scar density, she subsequently underwent iFAD with a 6.8 mm trephination at increased power at 160% and a 6.5 mm DMEK graft. The post-operative course was unremarkable. The DMEK graft remained attached with an overlying clear DALK at POM3. Vision improved to 20/50 (LogMAR 0.40).

### 3.2. Addressing the Issue of Both Deep Stromal Scarring and Endothelial Decompensation

#### 3.2.1. Patient 8

A 68-year-old male underwent a 7.00 mm manual optical DALK for stromal scarring secondary to a contact lens-related fungal infection. Subsequently, he developed a visually significant cataract for which he underwent phacoemulsification with a toric intraocular lens. His best corrected vision post cataract extraction was 20/100 (LogMAR 0.7). Clinically he was noted to have a clear graft with posterior stromal haze. There was also florid guttata present, with a central pachymetry value of 601 microns in the affected eye. He had subsequent worsening of vision in the affected eye to 20/200 (LogMAR 1.0), which was deemed secondary to endothelial decompensation. AS-OCT demonstrated posterior scarring with a thickened recipient stromal bed of 99–107 microns. As such, he underwent iFAD, with a 7.00 mm trephination at 150% power, followed by a 6.75 mm DMEK to remove both the endothelium and posterior stromal scars. BCVA at POM12 was 20/20 (LogMAR 0.0). ECC counts at the last review was 1838 cells/mm^2^.

#### 3.2.2. Case 9

A 57-year-old female with a history of bilateral advanced uveitic glaucoma (for which bilateral trabeculectomies were performed), herpetic epithelial keratopathy, punctate inner choroidopathy (PIC) and Fuch’s Endothelial Corneal Dystrophy (FECD) was treated for an acute episode of herpetic stromal melt in her affected right eye. Vision had reduced to counting-finger vision at 0.5 m. She underwent tectonic manual DALK which improved vision to 20/63 (LogMAR 0.5) up till post-operative year 4 (POY4). Following this, she presented acutely with a worsening vision to 20/200 (LogMAR 1.0). The cornea was hazy and edematous, with occasional pigments noted on the endothelium. A trial of oral antivirals and topical steroids failed to improve corneal clarity and vision, and vision was reduced further to counting fingers at 1 m, with an edematous overlying DALK. A diagnosis of endothelial failure was made, and she subsequently underwent a staged Ahmed Glaucoma Drainage Device, followed by iFAD with a trephination of 7.30 mm at 150% power, and a subsequent 7.00 mm DMEK. Post-operatively, DM detachment was noted, and she underwent rebubbling with 20% SF6. gas injection. The DMEK achieved stability subsequently, and POM9 BCVA improved to 20/40 (LogMAR 0.3).

## 4. Discussion

In this series, we demonstrate a surgical approach, “iFAD” to address deep stromal opacities or scars following DALK. Utilization of the liquid keratoplasty module on the Zeimer Z8 femtosecond laser system, combined with the benefits of iOCT during trephination, can work to deliver precise and consistent results, simplifying an otherwise complicated surgical problem.

Broadly, our cases can be divided into (1) those with remnant deep posterior lamellar folds or scars that affect the overall central visual acuity and (2) those with endothelial failure with some degree of overlying deep stromal scarring. It is important to note that all cases had manual ALK procedures performed prior to iFAD. A key advantage of a graded surgical approach (i.e., ALK, followed by iFAD as when needed), lies in the avoidance of the possible alternative—a PK at the outset. This is a significant clinical decision that can influence patient outcomes, as many of these cases are high-risk PK ones. Young patients, extensive stromal vascularization, or a history of immune or infective etiologies are all factors susceptible to greater lifetime risks of immunological graft failure where full-thickness transplants are performed [17]. As such, we advocate that these high-risk cases undergo an ALK as the initial procedure of choice [18]. Should remnant deep folds or scars persist after initial lamellar dissection, iFAD can then be utilized as a secondary procedure to optimize visual outcomes. Additionally, from a graft resource perspective, this stepwise approach to surgery can potentially enable eye banks to optimize graft utilization practices. Greater numbers of lower-grade corneas with reduced endothelial densities, that do not meet the criteria for EK-grade tissue, can be included for the purposes of the initial ALK [30].

No early or late graft rejections were reported in our series, which followed up with patients for a minimum of 1 year. We postulate that the reduction in total transplanted endothelial cells via a smaller DMEK graft size reduces the overall immunogenic trigger for graft rejection. This phenomenon is still poorly understood, and there is a paucity of reports in the currently available literature [17,18]. A systematic review conducted by Wu et al. looking at outcomes of DMEK or DSAEK following failed PK reported recipient age, graft size and previous glaucoma filtration surgery as the main risk factors for EK graft rejection under a failed PK. Still, in that review, pooled graft failure rates were 14% in patients that underwent DMEK following failed PK [32]. Arguably, the significantly reduced rates of graft rejection in our series could be explained by the fact that our patients underwent DALK as the initial surgery. Subsequent iFAD with DMEK could suggest that the rejection rates of these patients would be closer to that of a primary DMEK.

Regarding improvements in surgical technology, one major limitation in the early iterations of femtosecond-guided keratoplasties was the lack of real-time ability to plan and execute precise posterior host trephinations. While this is of minimal consequence for full-thickness trephinations, surgery can be made more difficult when addressing partial-thickness ones. The initial experiences described by Barrio et al. using the IntraLase^TM^ FS system (Intralase, Irvine, CA) required lesions to first be imaged and trephination depths estimated after studying separate AS-OCT scans [33,34]. A nominal side cut depth based on these estimations made in the clinic was subsequently programmed into the IntraLase^TM^ FS platform intraoperatively to perform the trephination. This produced trephinations of uniform depth across the cornea; however, it lacked versatility when non-uniform lesions or trephinations of varying depths were required. As a consequence, remnant stromal tissue bridges can result [34], requiring additional intraoperative manipulation. Our cases in comparison used iOCT throughout the docking, assessment and trephination phases. Trephination depths could be adjusted and set around a pre-determined diameter centred on the host cornea. (Figure 1) As such, variabilities in lesion depth could be accounted for during the final trephination. This is a significant advantage to the surgeon, as a more accurate fit of the posterior lamella can subsequently be easily dissected en-bloc bloc from remnant anterior stroma, despite having uneven side cut depths around the lamellae. In our cases, the only areas within the trephined lamella which were found to be relatively more adherent were old suture sites under the original ALK grafts. This was easily overcome intraoperatively by gentle plicating tractional forces applied onto the rest of the loosened lamellar sheet. Increasing trephination power up to 160% was also an effective strategy when dense stromal scars were encountered.

A separate challenge was the positioning of the reverse Sinskey hook when attempting to strip off the trephined posterior lamella. We found that it was crucial for the reverse Sinskey hook to be engaged within the femtosecond laser cut slot to allow the right plane to be subsequently dissected free. Engaging an incorrect plane anterior or posterior to this slot could result in uneven dissections or inadvertent scoring of the overlying stroma. This could lead to an uneven stromal surface, potentially portending higher rates of subsequent DMEK detachments.

The use of a liquid keratoplasty docking system afforded by the Z8 allowed geometric accuracy of trephinations, relative to other applanating systems. Applanation-based systems are known to cause extensive undulations in the DM following docking [23,24]. This flattening of the cornea has been shown to result in significant deformations across all layers of the cornea, especially in the deeper stromal and Descemets layers. Issues like inadvertent vertical tilt and geometric inconsistencies of the trephined lamella can result and have a bearing on subsequent stages of the surgery [24]. In the context of DMEK graft insertion, imprecise or inconsistent trephinations on the host cornea can result in size mismatches between the geometry of the trephined host and donor endothelium. This can affect the ability of the transplanted donor to maintain adhesion. Utilization of a liquid trephination interface minimizes compressive forces and distortion, preserving corneal dimensions and curvature throughout the entire trephination phase [24]. As such, vertical skewing during trephination can be kept to a minimum, and a predictable, congruently cut posterior trephination can be achieved. Another advantage of liquid-based docking systems is the reduction in collateral damage to endothelial cells adjacent to the trephination incision sites [20,24]. We found in our cases that in order to address a greater degree of light scatter in lesions with deep stromal scars, higher energy profiles were required to achieve effective degrees of trephination. Studies in porcine models have demonstrated significant differences in cellular denudement adjacent to trephination sites, with greater losses of mean adjacent cellular areas in applanation-based platforms compared to liquid-based ones [20,24]. These mean areas of cellular denudement increase correspondingly with the total percentage of power utilized for the trephination. Damage to endothelial cells has been found to be significantly greater using a flat applanation interface at 150% power, compared to corresponding liquid interfaces [24]. As such, the advantage of using a liquid interface is significant as a greater degree of collateral cell damage in the peripheral endothelium can have a long-term impact on future graft survivability [20].

In contrast to previously reported cases where DSAEK grafts were inserted following femtosecond laser-assisted endothelial keratoplasty, we chose in our series to perform DMEK for all cases. We believed that the advantage of performing DMEK was in the relative thickness of the graft, minimizing the probability of further interface issues following transplantation. Conversely, thicker DSAEK tissue can potentially lead to less-than-optimal visual outcomes due to the disparity in thickness between the central and peripheral cornea. Most of our cases achieved good post-operative BCVAs of between 20/20 to 20/50 (LogMAR 0.00–0.4). Cases which did not achieve vision at or better than 20/50 vision still had stable DMEK grafts that were attached during the last recorded visit. Case 6 achieved a final BCVA of 20/160, owing to pre-existing retinal pathology. The transplanted DMEK and cornea were clear at the last review. Performing the surgery with DMEK carries with it greater surgical complexity, as the graft needs to precisely fit within the boundaries created by the vertical trephination. Gas tamponade is also critical. Despite the use of 20% SF6 or air in pseudophakic cases or phakic cases, we experienced three cases of shallow detachments at the periphery of the graft on POD1, likely due to a tamponade issue. However, these cases were promptly treated with intracameral gas or air, which ultimately resulted in the resolution of the detachment.

A limitation of the current technology is its inability to perform a reproducible posterior horizontal lamellar trephination ‘de novo’ on the corneas that have posterior stromal scars. The reasons for this are the inherent difficulty overcoming the energy scatter whilst attempting to trephine the deep stroma, especially in cases where deep stromal scars are present. Additionally, bubbles from the opaque bubble layer (OBL) can migrate vertically, resulting in an irregular dissection plane [35]. Finally, the less compact nature of the deep stromal fibres relative to anterior fibres, and the potential undulatory nature of these deep astigmatic lesions often result in inconsistent trephinations, rough residual stromal beds and troublesome tissue bridges that require further plication intraoperatively [35]. Specific limitations of our study include the small case numbers and lack of long-term follow-up data. However, the purpose of this study is to demonstrate a surgical technique that can be applied to a variety of cases.

Femtosecond laser technology has seen great improvements in its availability, precision and predictability over the past two decades. Its applications have also since greatly expanded, serving not only as a useful adjunct to cataract surgery but also for a wide array of anterior segment and corneal procedures. In addition to its utility as an adjunct in improving surgical and visual outcomes in commonly performed keratoplasties, we have also demonstrated that in a variety of clinical indications, it can also serve as a powerful tool in helping to efficiently tackle an otherwise challenging surgical problem. As technological aspects of the femtosecond laser improve, however, the next exciting phase in femtosecond lasers’ contribution to deep lamellar keratoplasty would be through its ability to reliably create a horizontal posterior lamellar cut at any depth with minimal collateral damage to nearby endothelial cells.

## Figures and Tables

**Table 1:** Table of patient demographics, underlying pathology, and diameters before and after iFAD. Visual acuities before and after iFAD are in Snellen.

Case	Age	Sex	Underlying Pathology	VA Prior iFAD (Snellen)	Lens Status	Diameter Overlying DALK (mm)	Diameter iFAD Trephination (mm)	Diameter DMEK Graft (mm)	BCVA Post iFAD (Snellen)
1	72	F	HSK	20/160	Pseudophakic	8.00	7.60	7.50	20/30
2	55	M	HSK	20/160	Phakic	9.00	7.70	7.50	20/20
3	18	F	BKC	20/400	Phakic	7.75	7.40	7.00	20/30
4	83	M	GD	20/125	Pseudophakic	8.25	7.60	7.25	20/40
5	53	F	Infective Keratitis	CF 1.5 M	Phakic	7.75	7.50	7.25	20/25
6	82	M	IK	20/80	Pseudophakic	7.50	7.00	6.75	20/160
7	19	F	Stromal ectasia	20/200	Phakic	7.00	6.80	6.5	20/50
8	68	M	Infective Keratitis	20/200	Pseudophakic	7.50	7.00	6.75	20/20
9	57	F	HSK	CF 1 M	Pseudophakic	8.00	7.30	7.00	20/40

HSK—Herpetic Stromal Keratitis, BKC—Blepharokeratoconjunctivitis, GD—Granular Dystrophy, IK—Interstitial Keratitis.

## Data Availability

Data are contained within the article and Supplementary Materials.

## Supplementary Materials

The following supporting information can be downloaded at: https://www.mdpi.com/article/10.3390/bioengineering11121192/s1, Figure S1: Montage photographs of cases presented in manuscript. Cases 1–7 represent cases that had remnant deep stromal folds following anterior lamellar surgery. Cases 8 and 9 represent cases that had both deep stromal folds and endothelial decompensation. Figures S1a–S9a. Initial pathology of cases described in the manuscript. Figures S1b–S9b. Following anterior lamellar keratoplasty, with deep remnant stromal scarring resulting in suboptimal visual acuity. Figures S1c–S9c. Following iFAD strategy and subsequent DMEK graft insertion. Figure S2: Figures S2a–S2i. Montage anterior segment optical coherence tomography scans of cases in manuscript following initial anterior lamellar keratoplasty, demonstrating remnant deep stromal lesions.
