# Supplementary material for: Intraoperative Optical Coherence Tomography (OCT)-Guided Femtosecond Laser-Assisted Descemet Membrane Endothelial Keratoplasty (iFAD)
Source: Bioengineering (Basel). 2024 Nov 25;11(12):1192. doi: 10.3390/bioengineering11121192 (PMC11726893; doi:10.3390/bioengineering11121192)
Supplement: Supplementary file 1 [file bioengineering-11-01192-s001.zip › bioengineering-3300956-supplementary.pdf]

Supplementary Materials:

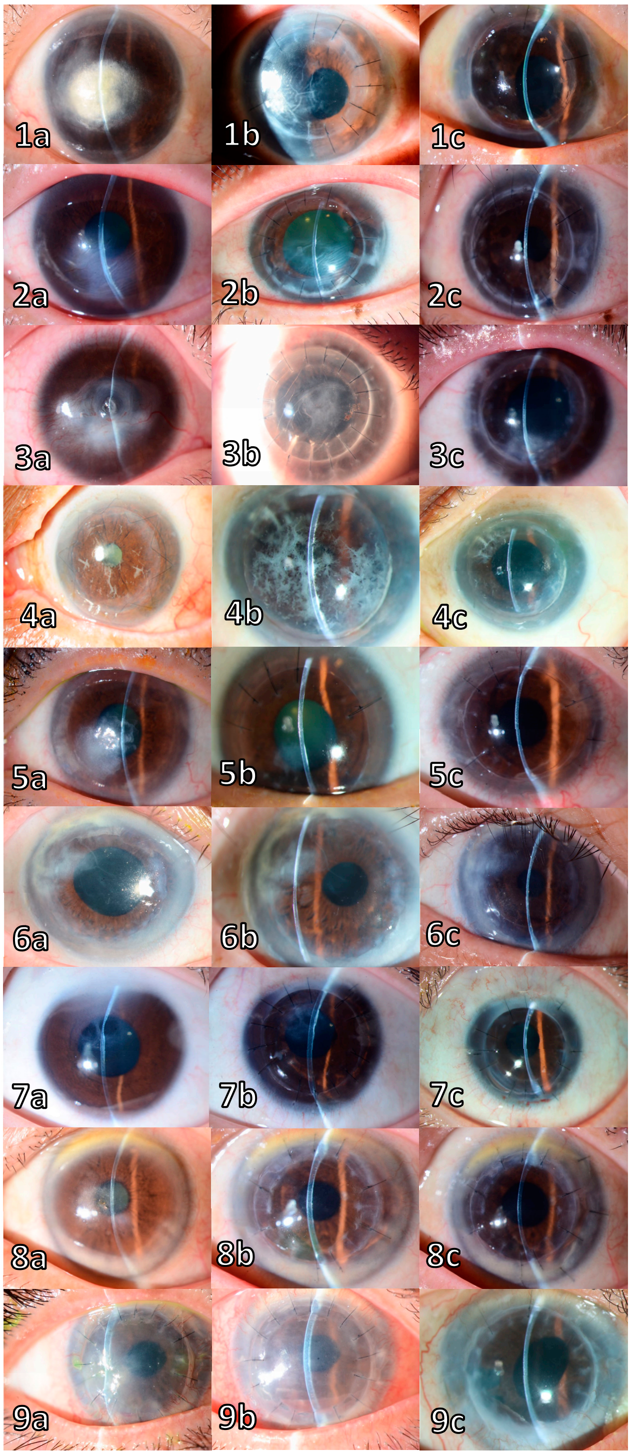

Figure S1. Montage photographs of cases presented in manuscript. Cases 1 – 7 represent cases that had remnant deep stromal folds following anterior lamellar surgery. Cases 8 and 9 represent cases that had both deep stromal folds and endothelial decompensation. Figures 1a – 9a. Initial pathology of cases described in the manuscript. Figures 1b – 9b. Following anterior lamellar keratoplasty, with deep remnant stromal scarring resulting in suboptimal visual acuity. Figures 1c – 9c. Following iFAD strategy and subsequent DMEK graft insertion.

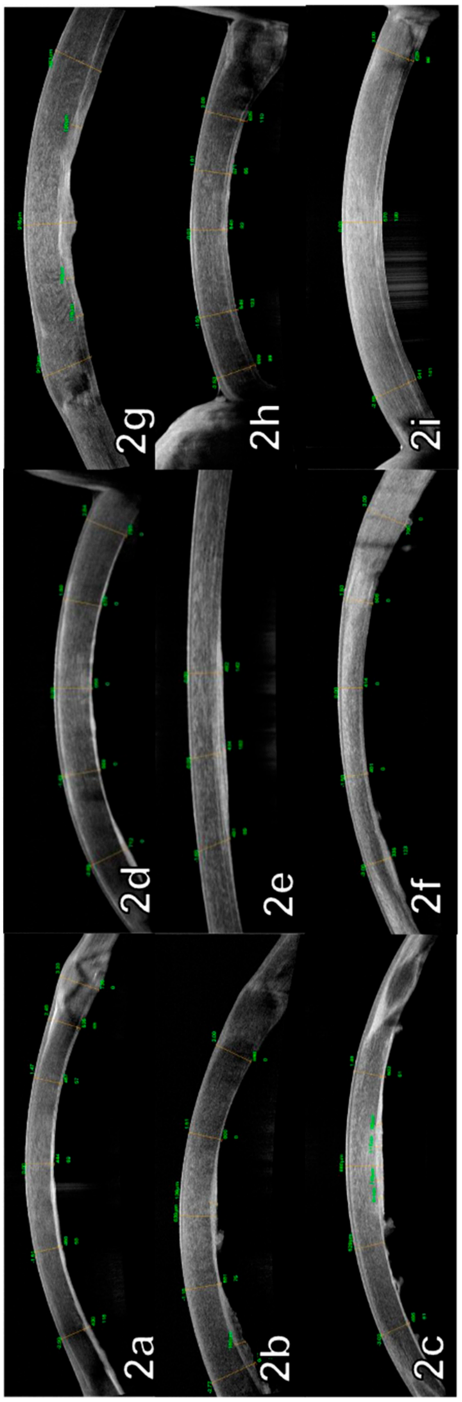

Figure S2. Figure 2a – 2i. Montage anterior segment optical coherence tomography scans of cases in manuscript following initial anterior lamellar keratoplasty, demonstrating remnant deep stromal lesions that required further treatment with iFAD.
